# Improving field establishment and yield in seed propagated Miscanthus through manipulating plug size, sowing date and seedling age

**DOI:** 10.3389/fpls.2023.1095838

**Published:** 2023-05-30

**Authors:** Chris Ashman, Rebecca Wilson, Michal Mos, John Clifton-Brown, Paul Robson

**Affiliations:** ^1^Institute of Biological, Environmental and Rural Sciences (IBERS), Aberystwyth University, Aberystwyth, United Kingdom; ^2^Energene Seeds Limited, Gogerddan, Aberystwyth University, Aberystwyth, United Kingdom; ^3^Terravesta Ltd, Lincoln, United Kingdom; ^4^Department of Agronomy and Plant Breeding I, Research Centre for Biosystems, Land-use and Nutrition (iFZ)), Justus Liebig University, Gießen, Germany

**Keywords:** bioenergy, energy crops, Miscanthus, establishment, biomass, glasshouse, perennial

## Abstract

Biomass crops provide significant potential to substitute for fossil fuels and mitigate against climate change. It is widely acknowledged that significant scale up of biomass crops is required to help reach net zero targets. *Miscanthus* is a leading biomass crop embodying many characteristics that make it a highly sustainable source of biomass but planted area remains low. *Miscanthus* is commonly propagated *via* rhizome, but efficient alternatives may increase uptake and help diversify the cultivated crop. Using seed-propagate plug plants of *Miscanthus* has several potential benefits such as improving propagation rates and scale up of plantations. Plugs also provide an opportunity to vary the time and conditions under protected growth, to achieve optimal plantlets before planting. We varied combinations of glasshouse growth period and field planting dates under UK temperate conditions, which demonstrated the special importance of planting date on yield, stem number and establishment rates of *Miscanthus*. We also propagated *Miscanthus* in four different commercial plug designs that contained different volumes of substrate, the resulting seedlings were planted at three different dates into field trials. In the glasshouse, plug design had significant effects on above and belowground biomass accumulation and at a later time point belowground growth was restricted in some plug designs. After subsequent growth in the field, plug design and planting date had a significant effect on yield. The effects of plug design on yield were no longer significant after a second growth season but planting date continued to have a significant effect. After the second growth year, it was found that planting date had a significant effect on surviving plants, with the mid-season planting producing higher survival rates over all plug types.Establishment was positively correlated with DM biomass produced in the first growth season. Sowing date had a significant effect on establishment but the impacts of plug design were more nuanced and were significant at later planting dates. We discuss the potential to use the flexibility afforded by seed propagation of plug plants to deliver significant impacts in achieving high yield and establishment of biomass crops during the critical first two years of growth.

## 1 Introduction

Biomass has significant potential to substitute for fossil fuels and contribute toward mitigating climate change (Creutzig et al., 2015). Many governments have issued directives in support for renewable energy from a range of sources including wind, solar, tidal and biomass. However, biomass has the potential to sequester carbon and provide not just energy but also bio renewable feedstocks for chemicals (Whitaker et al., 2018). The Committee for Climate Change (CCC, 2020) has reported that in the UK alone 23,000 ha per year of biomass crops are required to be planted until 2050 to help reach the UK government’s net zero goal. Despite this societal need, the planted areas of dedicated biomass crops remains relatively low due to a number of factors including uncertainty around prices and yield (Witzel and Finger, 2016).

One dedicated biomass crop that has received considerable attention due to high yields is the perennial C4 grass *Miscanthus* (Lewandowski et al., 2003; Heaton et al., 2010). Many studies of yield in *Miscanthus* have used the sterile triploid hybrid *M. × giganteus* (Hodkinson and Renvoize, 2001) (*M*×*g*). *Miscanthus* is planted in Spring and usually, in the first year, insufficient crop develops to make harvesting economical. In subsequent years yield increases to a maximum over 3-5 years and may be harvested from year 2 for 20-25 years (Lewandowski et al., 2003). While new varieties are being developed (Clifton-Brown et al., 2019) yields from currently available commercial varieties can be maximised by ensuring high crop establishment rates. Poor establishment, especially in perennial crops such as *Miscanthus*, can significantly reduce crop yields by lowering potential yield from having fewer plants per unit area and from slower progression toward maximum yield. Patchiness in *Miscanthus* crops, resulting from poor establishment, can significantly increase the time to repay initial investment and could reduce gross margins by more than 50% (Zimmermann et al., 2014; Dauber et al., 2015).

Yield is associated with latitude (Lesur et al., 2013) and at higher temperate latitudes economically harvested yield may not be achieved until year 3 making optimisation of planting especially important. On farm yield of *Miscanthus* was affected by a number of factors including establishment and weed competition (Lesur-Dumoulin et al., 2016) and the conditions of establishment were a significant factor in a multi-start trial (Tejera et al., 2019). If economical yield was achieved sooner or variability in establishment was reduced to provide a more predictable yield trajectory the adoption of *Miscanthus* crop production may be improved. Other approaches are being developed to provide farms with revenue in early years of *Miscanthus* cultivation such as co-cultivation with maize for anaerobic digestion (Von Cossel et al., 2019). However, such approaches cannot address the requirement for high levels of crop establishment which requires changes in agronomy to ensure a complete and competitive *Miscanthus* canopy is planted.

The most common method of propagating *Miscanthus* is *via* rhizome (McCalmont et al., 2017). When compared with propagation *via* seed, rhizome propagation slows adoption of new varieties, requires greater land for upscaling and thus represents a significant limitation to the rapid increase in plantation area (Clifton-Brown et al., 2017a; Ashman et al., 2018). Direct seed planting is unlikely to be viable in many areas due to thermal requirements for germination and resultant very low establishment rates (Hsu et al., 1985; Christian et al., 2005; Clifton-Brown et al., 2011). As an alternative, Miscanthus seed may be propagated through plug plants grown in controlled environments prior to planting in the field (Clifton-Brown et al., 2017a). Miscanthus plug plants deliver a number of benefits including an estimated cost saving of between 20-40% compared to rhizome propagation (Hastings et al., 2017).

The use of plugs to propagate *Miscanthus* from seed provides an opportunity to vary the length of time under protected growth and to vary environmental factors to optimise plantlets for subsequent growth and establishment in the field (Wu et al., 2022). One key aspect of *Miscanthus* production is the ability to establish plants effectively on less valuable land, reducing competition with arable crops (Campbell et al., 2008). Altering the treatments of plantlets under protected growth may improve competition with weeds and establishment of the crop upon subsequent transfer to the field. Time between sowing and planting of the plugs is one area that has great room for optimisation, as being able to plant seedlings of younger age and maturity would reduce establishment costs if survival and performance remain good. However, increasing seedling maturity through extending the glasshouse growth stage may positively influence establishment and therefore reduce gaps in the crop. Plug design also requires consideration, as a balance between numbers of plants produced per m^2^ and maximal biomass accumulation to allow for field survival needs to be considered. Restrictions imposed on the root system in smaller containers decreases the above ground dry weight accumulation (Di Benedetto and Klasman, 2004) and on average doubling the pot size can increase biomass production by 43% (Poorter et al., 2012). However, commercially grown plants are usually grown under restricted conditions due to the cost of greenhouse space and it is usually more profitable to produce higher numbers of plants in smaller modules to reduce the requirements for space and growth substrate. Exploration into the effectiveness of growing *Miscanthus* from seed is also ongoing in China; Zheng et al. (2021) reported poor performance with direct sowing, and subsequently experimented with growth in modules. Similar studies are required under more temperate climates such as the UK, for large scale roll out to growers, under a variety of conditions, and in a variety of climates.

This study aims to analyse survival and performance of *Miscanthus* plantlets after different glasshouse treatments. These include length of time spent in the glasshouse by altering sowing and planting timing, and glasshouse growth conditions by varying plug design. We hypothesised that extending the first year growth season of *Miscanthus* plantlets, in combination with optimised plug design, under a glasshouse environment would improve field establishment of the crop toward a higher and possibly earlier economic harvest. We varied planting dates in combination with glasshouse growth period to test the interaction of glasshouse and field growth periods. Having established the importance of planting date we examined the growth and establishment of *Miscanthus* seedlings planted at 3 different times in the field after glasshouse growth in 4 different commercial plug designs that contained different volumes of substrate.

## 2 Materials and methods

Results from two randomised field trials assessing agronomicand glasshouse treatments to aid establishment of seed based *Miscanthus* plug plants are reported. The trials assessed the effect of two glasshouse treatments contributing to plug development and maturity (sowing time and plug size) and one agronomic treatment (time of field planting) on development of *Miscanthus* in plugs plus subsequent field establishment and yield. The first field trial assessed the impacts of sowing date and planting date, the second field trial assessed the impacts of plug size and planting date. Both trials were planted by hand, planting depth varied with plug size all plugs were planted at a depth to ensure the entire root ball was covered by soil. After planting the plugs good root to soil contact was ensured by compressing the soil around the plug. Plots were rainfed and no fertiliser was applied following current best practice for the crop.

### 2.1 Sowing and planting dates trial

**Figure 1:**
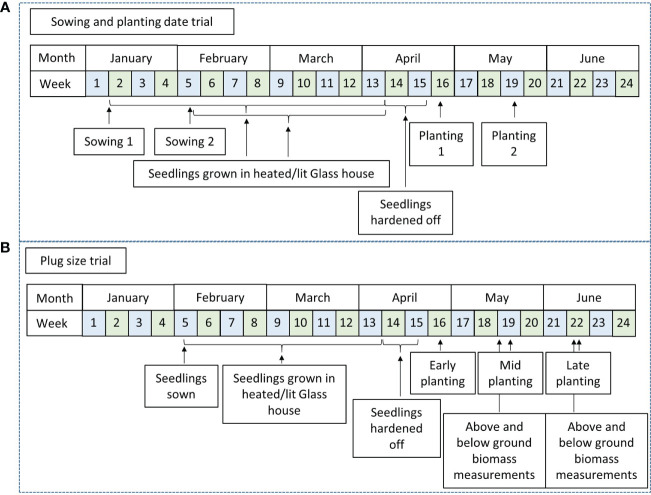
Timeline of key dates for planting and sowing of field trials to examine the effects of controlled environment treatments and field planting times on yield and establishment of Miscanthus plug plants from seed. **(A)** Sowing and planting date trial, **(B)** Plug size trial.

Treatments included two sowing dates (early and mid-season) and two planting dates (early and late). Sowing interval was four weeks, sowing 1 was early January (Jan. 6^th^) and sowing 2 early February (Feb. 2^nd^). Planting dates were late April (April 24^th^) and mid-May (May 16^th^) for planting 1 and planting 2 respectively (**Figure 1**). A Randomised Complete Block Design (RCBD) with three replicates for each treatment was used to test the four treatments 1) sowing 1-planting 1, 2) sowing 2-planting 1, 3) sowing 1-planting 2 and 4) sowing 2-planting 2. Plots contained 100 plants (10 rows x 10 plants) totalling an area of 66.66m^2^ per plot and were planted at a density of 15,000 plants ha^-1^.

### 2.2 Plug size and planting dates trial

Plugs were sown in early February, (Feb. 2^nd^). Three field trials were planted in an RCBD design, to take account of any field gradients, with three replicates of each treatment. Trials were planted throughout an expected planting season (early; 26^th^ Apr, mid; 17^th^ May and late; 14^th^ Jun) in 2017 to assess the impact of plug size and planting date on establishment and yield. Prior to the mid- and late-season planting, 10 randomly selected plants from a tray of each different type were removed and harvested. Each plug was cut just above the root mass of the seedling and above and below ground biomass were separated, roots washed and weighed individually. Samples were then dried to completion at 70°C and re-weighed. Plot size varied between planting dates. Plots planted at the early planting contained 100 plants (10x10) totalling 75.2 m^2^ per plot whilst the mid- and late-plantings contained plots of 50 plants (5x10) totalling 37.6m^2^ per plot. Planting density was 13,333 plants ha^-1^ for all planting dates.

**Figure 2:**
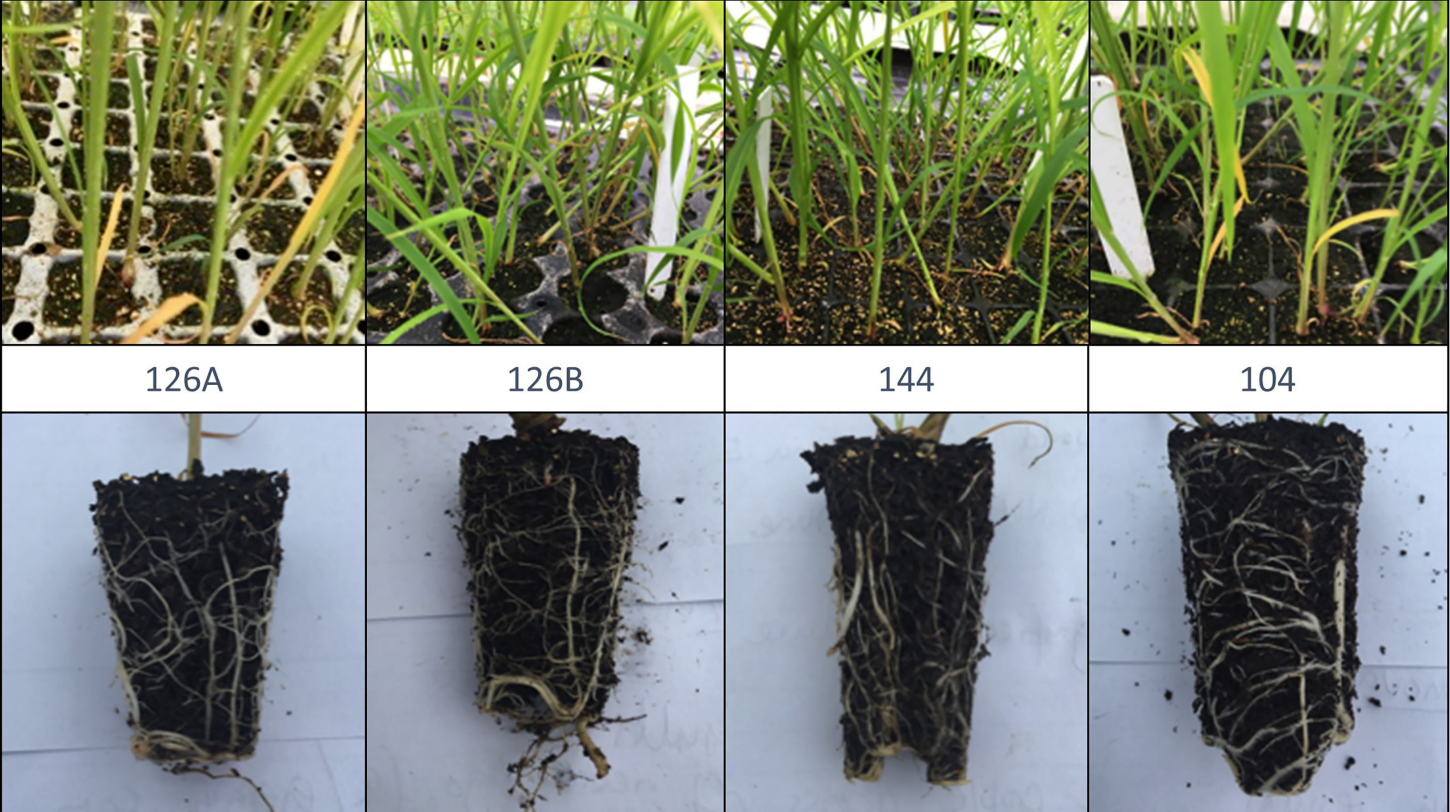
Images showing plantlet density and root growth in exemplars from four different module tray plug designs (for dimensions and volumes see **Table 1**).

Four disposable module trays with varying numbers of cells per tray, different cell depths and cell volumes were tested. These were designated by the number of modules being; 104, 126 and 144; two trays contained 126 modules but with differing plug volumes designated as 126 A, 126 B, (**Figure 2**). Plug volumes were 25 cm^3^, 35 cm^3^, 45 cm^3^ and 70 cm^3^ for 126 A, 126 B, 144 and 104 respectively (**Table 1**, **Figure 2**). To evaluate the relative costs incurred for each of the module types, cost efficiency was calculated using the variables required to grow the plugs. These included costs of glasshouse space and the associated requirements (supplemental light and heat), compost volume and tray price.

**Table 1:** Plug module information and parameters for each of the four plug sizes used in this experiment.

Plug module type	126A	126B	144	104
Volume of soil (cm^3^)	25	35	45	70
Depth (cm)	4	5	5	5.5
Soil capacity (g)	8.5	11.9	15.3	22
Trays per 100m^2^ of glasshouse	63	63	61	61
Plants per 100m^2^ of glasshouse	7925	7925	8764	6330
Relative price per plug plant (*, lowest cost; **, mid range; ***, highest cost)	**	**	*	***

### 2.3 Plug production

Plugs were produced by Bell Brothers Nursery ltd, (Boston, Lincs, UK), a commercial nursery specialising in modular production of bedding plants. Commercial protocol was followed, adding 2 seeds per cell, to ensure a minimum of empty plugs. Sowing dates varied from early January to late February (**Figure 1**). Ambient growing conditions at this time of year were unfavourable for plug growth and supplementary light and heat was provided to aid plug development. Sodium lights were used at both ends of the photoperiod to extend this to 12h and aiming to provide around an average of 150 moles m^-2^ d^-1^. Ambient glasshouse temperatures were between 20-25°C and 15-20°C during day and night respectively. In early April seedlings were moved to an unlit cooler glasshouse to harden off prior to planting. A promising seed based inter-specific hybrid, GNT 14, bred at IBERS (Institute of Biological, Environmental and Rural Sciences), was used in the trials. GNT14 is a hybrid of *Miscanthus sinensis* and *Miscanthus sacchariflorus* and was chosen for its high yield and homogeneity.

### 2.4 Land preparation and location

Field trials were established at Hackthorn, Lincolnshire (53°19’50.8”N, 0°28’11.7”W) in 2017 in collaboration with Terravesta ltd (Lincs., UK). The trials were located in a field previously used for arable rotation. Soil texture (0-30cm homogenised) was silty clay and is representative of lower grade land (grade 3-4) that is likely to be favoured for biomass production. Trial areas were ploughed in the autumn before planting following standard agronomic practices for arable crop production on this land type. Prior to planting, the trial areas were sprayed with a glyphosate-based herbicide to remove any weeds and cultivated with a power harrow to provide a tilth suitable for plug planting.

Plants were covered with Samco ‘grey’ mulch film, (Samco ltd, Adare, Co. Limerick, Ireland) immediately after planting. The mulch film remained on the plot until it degraded approximately 6 weeks after planting.

A pre-emergent herbicide mix of Stomp aqua (1.5 l ha^-1^) (BASF) and Callisto (1.5 l ha^-1^) (Syngenta) was applied after planting but prior to covering plots with mulch film. In year one any weeds were removed from trial areas by strimming once the mulch film had degraded.

### 2.5 Harvest yield quantification and phenotypic measurements

Plant establishment rates were estimated after the first growth season (September 2017) and overwintering survival after the first winter, once surviving plants were growing during the subsequent summer (August 2018). All surviving plants were counted and establishment and overwintering expressed as a percentage of the total planted per plot.

Phenotypic traits and yield were measured in year one and year two of both trials. Edge rows were disregarded from measurements. Phenotypic traits: stem count, stem height and canopy height were measured in autumn using eight plants per plot from the central row of the harvest quadrat. Stem height was measured to the top ligule of the tallest stem on each plant. Stem count included all stems deemed to contribute to the plant canopy, defined as all stems greater than 50% height of the tallest stem.

Yield was calculated from a quadrat harvest taken in late winter (February/March) in 2018 (year 1 growth) and 2019 (year 2 growth). 24 plants were harvested by hand and the material was weighed using a hanging balance and tripod. Quadrats ranged from 16m^2^ to 18m^2^ (max 24 (3 X 8) plants) and were taken from the centres of plots. Sub samples of ~200g were collected from each quadrat and dried at 105°C to constant weight to assess moisture content at harvest and calculate dry matter yield (DM t ha^-1^). Because impacts on establishment were reported separately, if plants were missing from the quadrat, yield was adjusted as a proportion of the measured yield to represent a crop at 100% establishment.

### 2.6 Data analysis

All data were analysed using R version 3.6.3 (2020-02-29) (R Core Team, 2020). Data normality and equality of variance were tested using Shapiro-Wilkes and Levene’s tests to determine if parametric or non-parametric methods were used to compare means. Where significant differences of means were detected by ANOVA results are reported in text including F and P values in parentheses. *Post-hoc* analysis used TukeysHSD function from Agricolae (Mendiburu, 2020).

## 3 Results

### 3.1 Interactions between sowing and planting dates on establishment in *Miscanthus*

To examine the potential to enhance field growth and survival of *Miscanthus*, seedlings were sown into plugs at two different dates, grown in the glasshouse and planted in the field at two different dates. Dry weight yield and stem morphology were measured in the resulting plants after two subsequent growth seasons and establishment rates after one growth season (**Table 2**). There was a marginally significant effect of planting date on the percentage of plants establishing in the field [F_1,9 =_ 5.4, p=5x10^-2^] but not sowing date. Establishment rates were lower after the second planting date irrespective of sowing date. The percentage of plants established from sowing dates 1 and 2 was 96.7 ± 2.4 and 95.7 ± 3.8 respectively when planted earlier in the year whereas these rates fell to 86.7 ± 1.3 and 90.7 ± 4.7% respectively after the later planting.

**Table 2:** Establishment, yield and stem traits after two growth years of *Miscanthus* propagated as seedlings for different times in the glasshouse and sown in the field at different dates.

Sowing date	06/01/17	06/01/17	02/02/17	02/02/17
Planting date	24/04/17	15/06/17	24/04/17	15/06/17
Establishment (%)	96.7 ± 2.4 (a)	86.7 ± 1.3 (a)	95.7 ± 3.8 (a)	90.7 ± 4.7 (a)
Overwintering (%)	96.0 ± 2.3 (a)	90 ± 3.1 (a)	98.7 ± 0.9 (a)	90.3 ± 4.4 (a)
DW (2018) (t Ha^-1^)	4.6 ± 0.4 (a)	1.8 ± 0.4 (b)	6.1 ± 0.6 (a)	1.6 ± 0.6 (b)
DW (2019) (t Ha^-1^)	10.7 ± 1.2 (a)	7.4 ± 0.1 (a)	9.4 ± 0.3 (a)	8.5 ± 1.2 (a)
Stem height (2017) (cm)	96.7 ± 11.4 (a)	49.2 ± 1.7 (b)	121.5 ± 3.4 (a)	47.7 ± 8.8 (b)
Stem height (2018) (cm)	161 ± 13.1 (a)	120.3 ± 11.7 (a)	151.2 ± 10.4 (a)	101.4 ± 21.0 (a)
Stem count (2017)	29.6 ± 3.5 (ab)	19.1 ± 4.4 (bc)	35.8 ± 2.3 (a)	13.6 ± 3.0 (c)
Stem count (2018)	31.7 ± 5.1 (a)	29.2 ± 4.8 (a)	34.3 ± 2.6 (a)	29.8 ± 4.9 (a)

Values are means ± standard errors (n = 3). Letters in parenthesis indicate significant groups, within traits (rows), identified by a Tukeys *post-hoc* test.

There was a significant effect of planting date on the percentage of plants that survived overwinter and grew in the second season [F_1,9 =_ 6.5, p=3x10^-2^] but not sowing date. Trends in overwintering rates were similar to those in establishment being lower after the second planting date irrespective of sowing date suggesting if plants established in the first season they were able to overwinter. A small number of plants were not scored at establishment but were scored after overwintering which may be due to aboveground growth being lost in year 1 while sufficient belowground biomass remained to initiate growth after winter.

Harvested yield was significantly affected by planting date [F_1,20 _= 27.8, p=3.7x10^-5^] and year of harvest [F_1,20 _= 99.9, p=3.2x10^-9^] but not sowing date and there was no significant interaction between factors. When harvests from sowing dates were combined, after the first year (growth year 2017, harvested Spring 2018), plants grown from planting date 1 produced 5.4 ± 0.5 DM t ha^-1^ and from planting date 2 produced 1.7 ± 0.3 DM t ha^-1^. This difference was less after the second growth year (growth year 2018, harvested Spring 2019) when harvested yields of 10.0 ± 0.6 DM t ha^-1^ and 7.9 ± 0.6 DM t ha^-1^ were produced from planting dates 1 and 2 respectively.

When stem morphology was measured after the first and second growth years there was a significant effect of planting date (F_1,20 _= 39.9, p=3.7x10^-6^) and growth year (F_1,20 _= 42.6, 2.3 x10^-6^) on stem height but no significant effect of sowing date. When years were examined separately any effects of glasshouse sowing date remained insignificant. The trends in stem number were similar in that there was a significant effect of planting date and growth year but there was also a significant interaction (F_1,16 _= 5.219, p=3.6x10^-2^) because stem numbers were similar across all treatment combinations except treatments including the second planting date. Fewer stems developed in the first growth year after both later planting treatments (sowing dates 1 and 2) but numbers recovered to similar levels across all treatment combinations after the second growth year (**Table 2**).

### 3.2 Effects of plug design on growth of *Miscanthus* seedlings under glasshouse conditions

**Figure 3:**
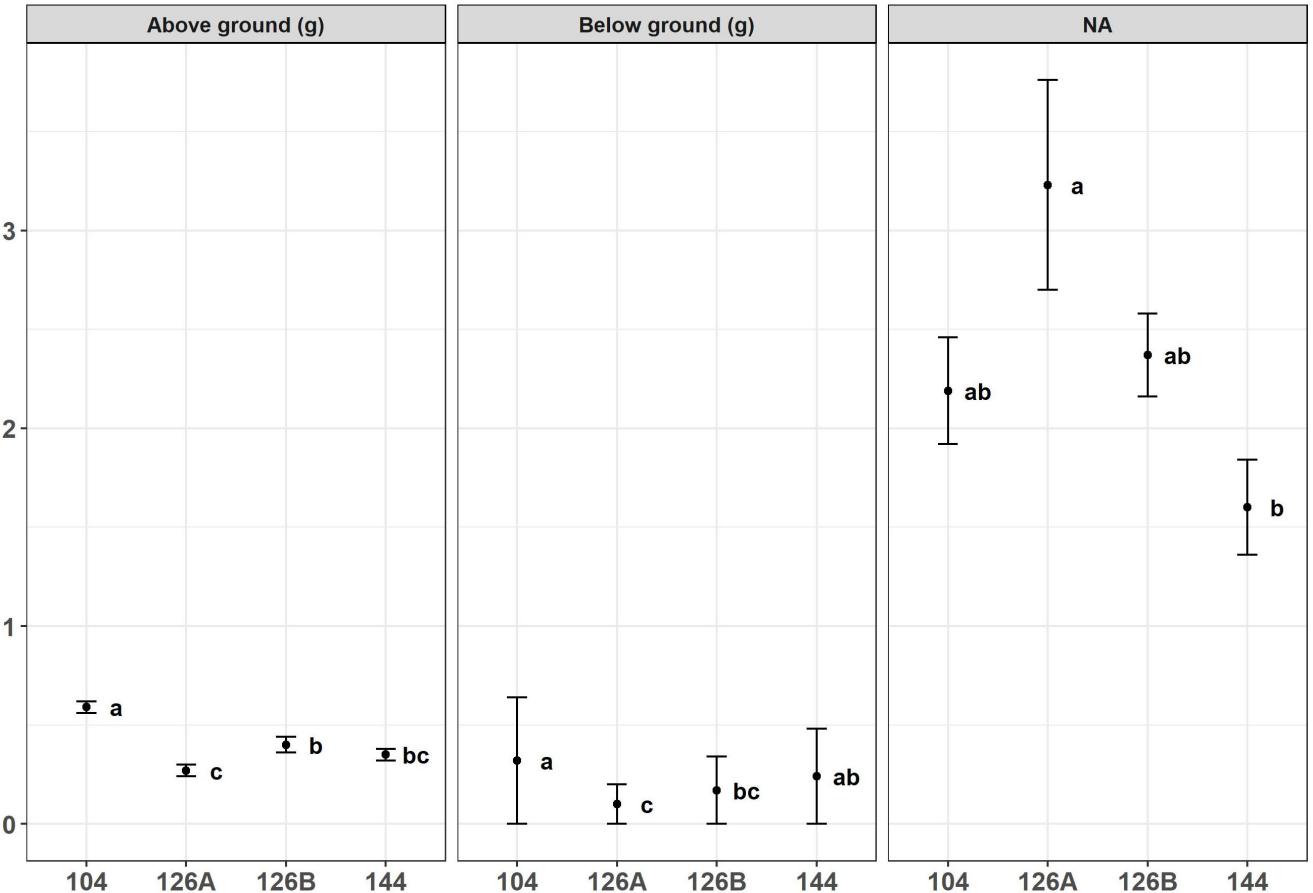
Above ground biomass (g), bellow ground biomass (g) and aboveground: belowground biomass ratio produced by *Miscanthus* seedlings growing in four different plug designs under glasshouse conditions prior to field planting. Error bars show SE, n=10. Letters represent significant *post hoc* groupings examined within 4 plug sizes measured in May.

**Figure 4:**
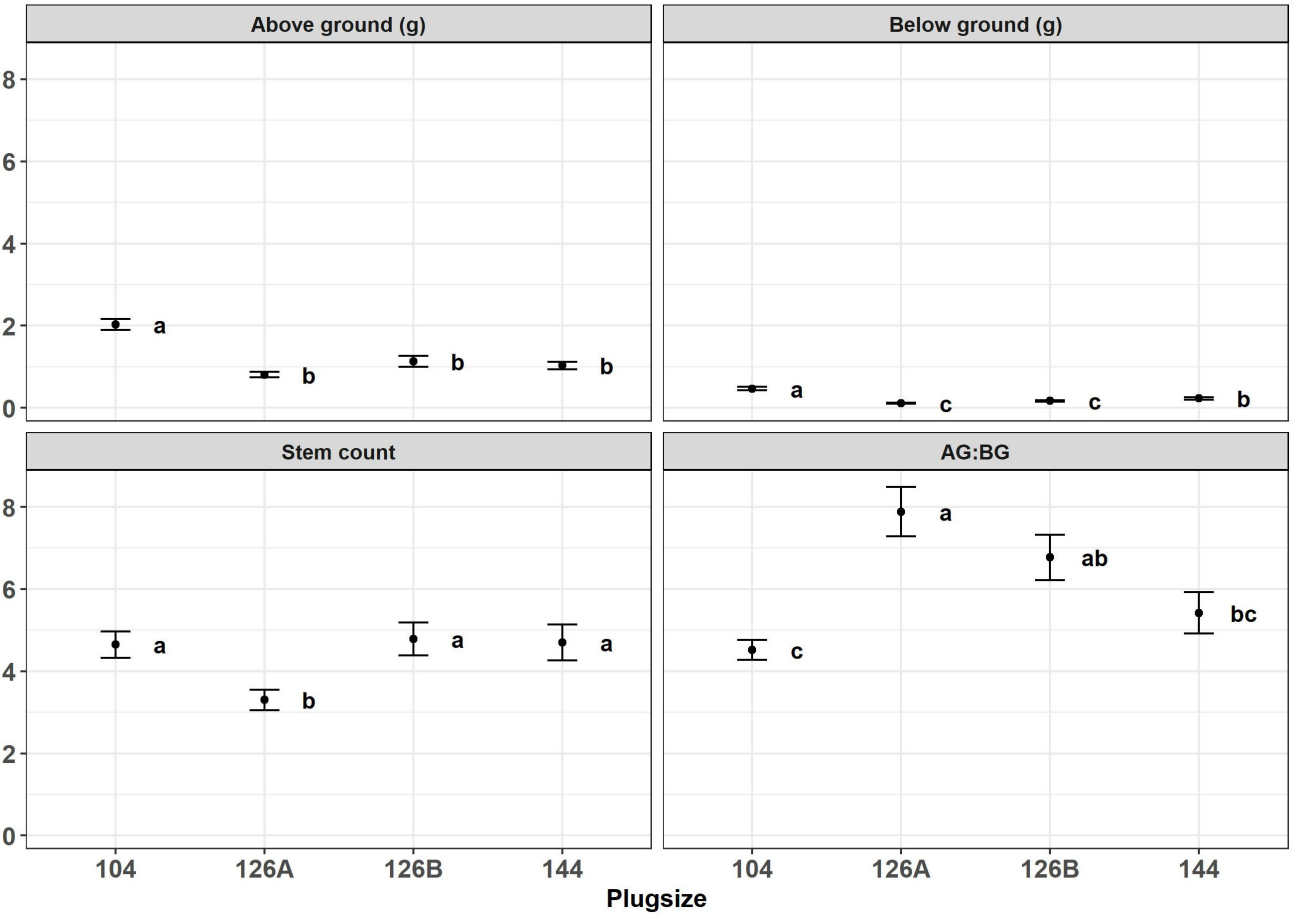
Above ground biomass (g), bellow ground biomass (g), stem count and aboveground: belowground biomass ratio produced by *Miscanthus* seedlings growing in different plug designs under glasshouse conditions prior to field planting. Error bars show SE, n=20. Letters represent significant *post hoc* groupings examined within 4 plug sizes measured in June.

Growth of *Miscanthus* plants in four different plug types was monitored during the period of glasshouse growth prior to planting in the field. There was a significant effect of plug type on above- and below-ground dry weight of biomass from *Miscanthus* plants growing under glasshouse conditions at the two sample dates May and June (**Figures 3**, **4**). For example, aboveground dry weight at the May sample date [F_3,36_ = 6.0, p=1.9x10^-3^] was significantly larger in seedlings growing in the 104 plug type. Plug type 126B produced the next largest plants which were significantly larger than plants from plug type 126A which produced the least biomass. Plants growing in plug type 144 were in the middle and overlapped with biomass values from plants growing in plug types 126B and 126A resulting in the following rank order of biomass for plug type 104>126B>144>126A. Irrespective of biomass measurement, aboveground or belowground, the rank orders were similar in that plants growing in plug type 104 were always the highest and were always significantly different to the plants growing in plug type 126A which produced the lowest biomass values. The other two plug types produced intermediate values of biomass that were not significantly different. The ratio of aboveground to belowground biomass significantly increased between the May (grand mean = 2.32 ± 0.18) and June (grand mean = 6.15 ± 0.28) sampling dates [F_1,111 _= 100.5, p=<2.0x10^-16^], which resulted from a significant increase in aboveground biomass. Belowground biomass did not change significantly between the two sample dates [F_1,111 _= 2.8, p=9.5x10^-2^]. Pairwise comparison indicated belowground biomass only increased significantly between the two sample points (May and June) in plants grown in plug type 104 [p=2.5x10^-2^], belowground biomass was relatively unchanged in plants growing in all other plug types. Additional morphologies were measured at the June glasshouse sampling date. Plug design did not have a significant effect on either elongation of the longest stem to the youngest ligule or canopy height (data not shown) but stem number was significantly different [F_3,75 _= 3.8, p=1.3x10^-2^]. Plug type126A produced plants with significantly fewer stems (3.30 ± 0.25) whereas all other plug types produced similar numbers that averaged closer to 5 stems (**Figure 4**).

### 3.3 Interactions between plug design and planting dates on establishment in *Miscanthus*

**Figure 5:**
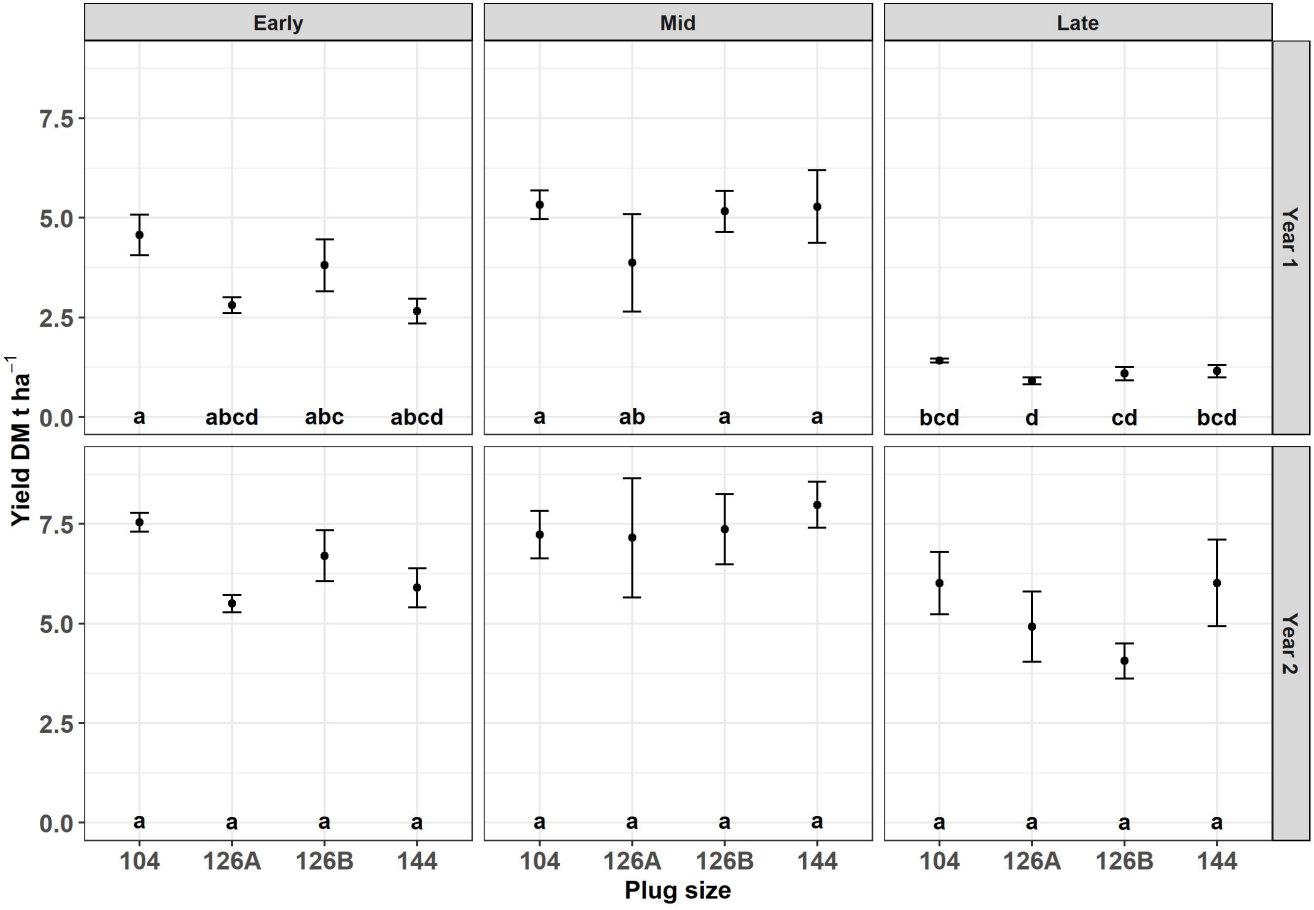
Harvest yield after year 1 and year 2 of *Miscanthus* established from plug plants propagated in glasshouses in four different modules sizes and planted at three different planting dates early (April), Mid (May) and late (June). Error bars so SE (n=3). Letters represent significant *post hoc* groupings examined within 4 plug sizes.

The plants grown in four different plug types were planted at three different dates, grown under field conditions and monitored over two growth years. Block did not have a significant effect on DM yield of the harvested crop and therefore blocks were removed as a factor from further analyses. There was no significant interaction between the remaining three factors. There was a significant effect of growth year (F_1,48 _= 136.3, p = 1.3x10^-15^), planting date (F_2,48 _= 39.7, p < 6.7x10^-11^) and plug type (F_3,48 _= 3.0, p = 3.9x10^-2^) on DM yield. Biomass harvested after the first and second growth years was examined separately and there was no significant interaction between plug size and planting date in either year, so only the main effects were examined. After the first growth year there was a marginal effect of plug type (F_3,30 _= 2.9, p = 5.0x10^-2^) but a significant effect of planting date (F_2,30 _= 50.4, p = 2.5x10^-10^) on DM yield. *Post hoc* analysis indicated that the DM yields after one growth year of plants originally grown in plug types 104 and 126A were significantly different, producing the highest and lowest amounts of biomass (3.8 ± 0.6 DM t ha^-1^ and 2.5 ± 0.6 DM t ha^-1^ respectively) the other two plug types resulted in intermediary amounts of biomass and were not significantly different from each other or from the two extremes. *Post-hoc* analysis indicated that DM yield was significantly different from all three planting dates. The DM yield after one growth year, averaged over all plug types, was highest after the mid planting date (4.9 ± 0.4 DM t ha^-1^), lowest after the late planting date (1.1 ± 0.1 DM t ha^-1^) and the early planting date was intermediate (3.5 ± 0.3 DM t ha^-1^) (**Figure 5**).

After the second growth year the effect of planting date continued to have a significant effect on DM yield (F_2,30 _= 8.0, p = 1.7x10^-3^) but plug type was no longer a significant factor although plants grown from plug types 104 and 126A continued to produce the highest and lowest amounts of biomass respectively. The relative order of yields produced by plants planted at different times (mid>early>late) remained consistent between years 1 and 2; however, the difference between the highest biomass yield from the mid planting (7.4 ± 0.4 DM t ha^-1^) and the early planting (6.4 ± 0.3 DM t ha^-1^) was no longer significant.

**Figure 6:**
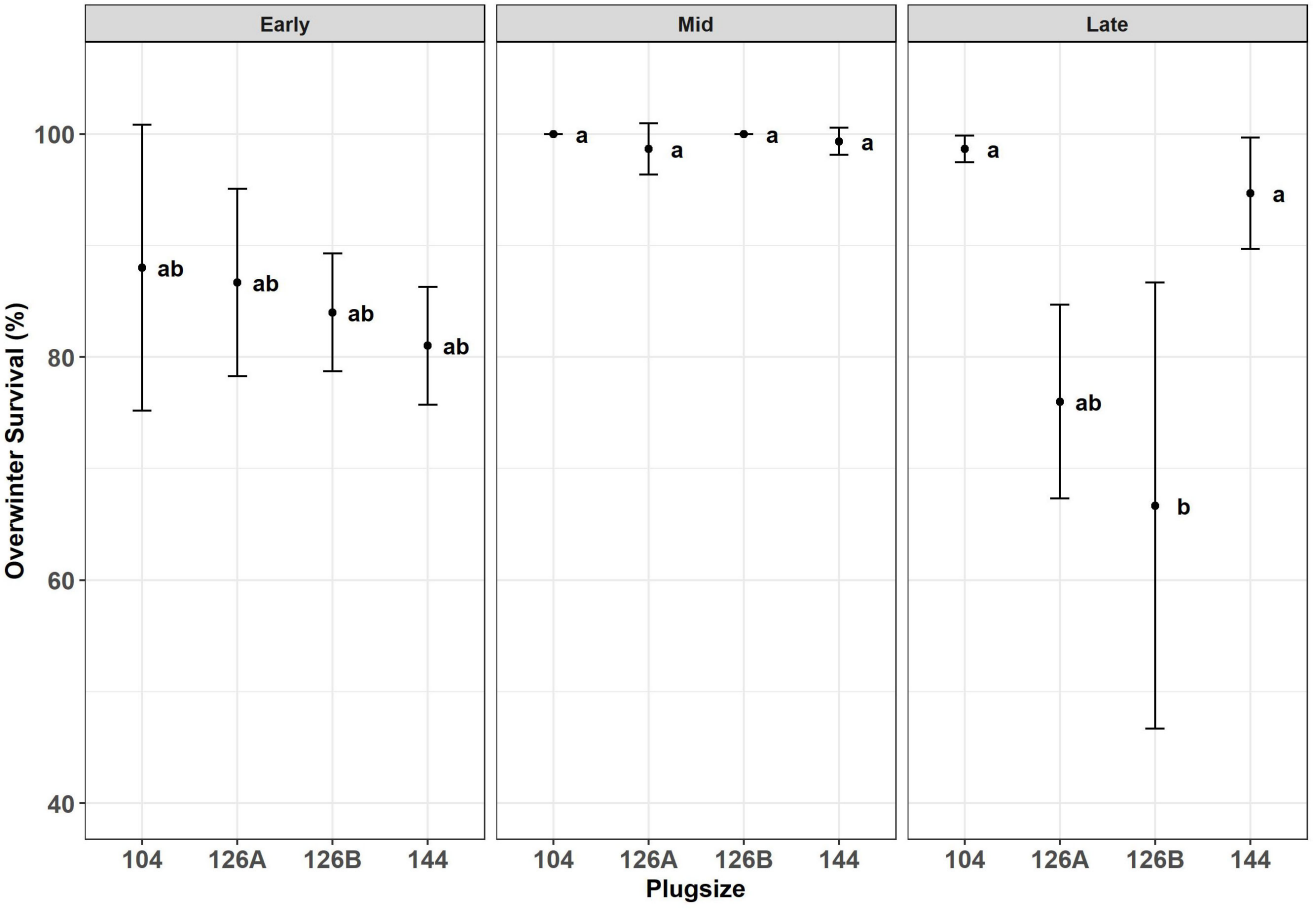
Overwinter survival after the second years growth of *Miscanthus* established from plug plants propagated in glasshouses in four different modules sizes and planted at three different planting dates early (April), Mid (May) and late (June). Error bars so SE (n=3). Letters represent significant *post hoc* groupings examined within 4 plug sizes.

Surviving plants were counted during the second growth year (August 8^th^, 2018) (**Figure 6**). Block did not have a significant effect on survival and therefore was removed as a factor from further analyses. Planting date had a significant effect on the percentage of plants surviving (F_2,30 _= 18.7, p = 8.7x10^-5^); combining all plug types the mid planting date produced significantly higher mean survival (99.5% ± 0.4), than either early or late plantings (84.9 ± 2.2 and 84.0 ± 4.8 respectively). Plug type did not have a significant effect on survival over all data but when planting dates were examined separately, plug type had a significant effect on survival rate at the late planting date only (F_3,8 _= 8.6, p = 3.5x10^-2^). Within the late sown plants plug type 104 and 144 had the highest survival rate (98.7 ± 0.7 and 94.7 ± 2.9 respectively); whereas plug types 126A and 126B the lowest (76.0 ± 5.0 and 66.7 ± 11.6 respectively) (**Figure 6**). Survival at Autumn 2018 was positively correlated with DM biomass produced in the first growth year and harvested in the previous Spring (2018) (R = -0.59; p = 4.0x10^-2^).

Planting date had a significant effect on stem number and height in the first and second Autumn after planting (F_2,30 _= 33.683; p = 2.14x10^-8^ (2017); F_2,30 =_ 11.816; p = 1.6x10^-4^ (2018) for stem number; F_2,30 _= 79.850; p = 9.7x10^-13^ (2017), F_2,30 =_ 8.377; p = 1.3x10^-3^ (2018) for stem height). At the first Autumn assessment (2017) the plants from mid planting date always produced the most (35.5 ± 1.8) and longest (144 ± 4.7 cm) stems. Plants from the late planting date produced the fewest (18.8 ± 1.3) and shortest (72 ± 3.2 cm) stems and plants from the early planting date were intermediate (26.1 ± 1.6 stems and 118.1 ± 4.6 cm stem height). The trends between plants from the three planting dates were sustained through the next growth season to at least the second Autumn assessment (**Table 3**).

**Table 3:** Stem traits per plant after the first and second growth years of *Miscanthus* propagated as seedlings in four module sizes (data combined) in the glasshouse and sown in the field at three different dates.

Growth year	Planting	Stem count per plant	Stem height (cm)
Year 1	Early	26.1 ± 1.6 (b)	118.2 ± 4.6 (b)
Year 1	Mid	35.5 ± 1.8 (a)	144.8 ± 4.7 (a)
Year 1	Late	18.8 ± 1.3 (c)	72.0 ± 3.2 (c)
Year 2	Early	26.8 ± 1.3 (a)	104.3 ± 4.4 (ab)
Year 2	Mid	21.4 ± 1.4 (b)	120.1 ± 4.4 (a)
Year 2	Late	17.4 ± 1.5 (b)	88.6 ± 8.9 (b)

Values are means ± standard error, (n = 12). Letters in parenthesis indicate significant groups, within years and traits, identified by a Tukeys *post-hoc* test.

## 4 Discussion

To fully exploit the potential for large-scale roll-out of seed-based hybrids of *Miscanthus* innovations in *Miscanthus* propagation and plug planting methods are needed (Clifton‐Brown et al., 2017a). Many factors could affect establishment and early growth of *Miscanthus* plug plants under field conditions, including genotype, seed size, soil characteristics, meteorological conditions and seedling age at planting. Some of these factors are not controllable, others require extensive time and effort for research and breeding; however, manipulating the growth of plants prior to field planting represent an adaptable flexible approach that can be applied to the crop immediately. The growth of biomass crops such as *Miscanthus* is being targeted toward areas of low grade land that is economically unfeasible for food production, thus reducing potential competition with food crops (Valentine et al., 2012). Therefore, in these experiments we utilised low grade land under temperate conditions and examined how different glasshouse treatments impacted growth and establishment of the crop.

According to the ‘reserve effect’, during the initial growth period larger seeds or seedlings, which contain more reserves, can support plant growth and repair any damage sustained under less optimal conditions (Westoby et al., 1996). *Miscanthus* produces small seed (Clifton-Brown et al., 2017b) and therefore we hypothesised that a longer period of growth in the glasshouse or growth in larger substrate volumes will ideally produce a large biomass seedling better able to survive and develop under field conditions after plantation.

### 4.1 Module design

*Miscanthus* plug planting is a relatively new innovation and the module of choice until the current study has typically been a 25cm^3^, 4cm deep module in trays of 126 individuals. These plugs have small volumes but provide the ability to produce large quantities of plugs in a small area. This was one of the four module sizes tested in the plug sizes assessment. Other module designs tested increased the volume and depth of substrate and the largest (104) had just under three times the volume of the original design and a rooting depth of 5.5cm but a concomitant reduction in the density of plantlets. In agreement with our findings a similar study concluded that using larger plug volumes led to inefficient use of substrate and glasshouse space, whereas using smaller plug volumes led to underdeveloped, weak plants (Zheng et al., 2021). The largest plug size (104 with 70cm^3^ soil volume) provided comparable growth and vigour with the next size down, but with a production deficit of 28% per unit area, whereas the smallest size (126A with 25cm^3^ soil volume) had a negative impact on performance and crucially, survival in the first year. The 144 modules (45cm^3^ soil volume) allowed a balance to be found between production per unit area of available glasshouse space, and strong, fast growing plugs with flexibility in time spent under glasshouse conditions.

Although all plug types represent a very small volume of growing media compared to conventional plant pots it is interesting to compare with a meta-analysis of the impacts of pot-sizes that demonstrated the main effects limiting growth were from reduced photosynthesis rather than morphology or resource allocation (Poorter et al., 2012). We did not measure photosynthesis but demonstrated that in all but the plug type with the largest soil volume (104) root growth was static between two time points sampled. Aboveground growth continued to increase during this period resulting in increasing aboveground: belowground ratios including plants growing in plug type 104 where root growth continued to increase. Root binding could cause reduction in establishment when planting out into free soil. In species of pine, tree survival and growth after planting is directly related to the ability of the root system to rapidly colonise and grow into the surrounding soil (Schultz and Thompson, 1997). With the results of the late planting here, it is probable that the lack of free growing roots reduced the root growth potential when planted, reducing the seedling’s ability to deal with water stress after planting (McTague and Tinus, 1996).

### 4.2 Conditions at planting

The ‘sweet spot’ in planting time will be transient and dependent on weather conditions over the season. In the module size trials reported here, the early season planting was at the end of April. Weather assessments of the weeks leading up to, and the days following the planting revealed that the planting took place in a moderate dip in air temperature at the time to around 4°C on average but rising to approximately 12°C over the next week. The rainfall was low beforehand, but moderate at the time of planting and over the next couple of days, and the survival of this trial was good.

During the mid-season module planting in mid-May, average temperatures were higher at around 15°C and there was moderate rainfall before, during and after the planting. The survival rate was 100% over the first few months (data not shown) for all plug modules, suggesting the weather and environment conditions at the time were optimal.

The late season module planting in mid-June was planted during a dry spell, with no rain the week leading up to planting, or after planting for approximately 8 days. Temperatures were higher on average, with a spike of temperature reaching 24°C on average a few days later, before reducing again in later June. These variable weather conditions appear to have a significantly negative effect on plant survival, particularly in the smaller plug sizes. The warm and dry conditions of the later planting could potentially have had an especially negative effect on the plants under film, as temperatures can rise rapidly in direct sunshine under film (Ashman et al., 2018). In addition to the impact of meteorological conditions at planting, we suggest smaller plugs, when planted later were disadvantaged compared with larger seedlings. Under the resulting shortened growth season, larger plants with greater maturity and resilience have a greater head start toward producing sufficient biomass to survive over winter.

### 4.3 Performance and yield

The impacts of plug type on biomass harvested in the field were relatively short-lived and had a significant effect after the first growth year (**Figure 5**). Although the larger plugs continued to produce more harvested biomass the differences were no longer significant after 2 growth years. Ultimately it would be expected that any differences in biomass of the crop will disappear as the crop approaches peak yield and/or a highly competitive and complete canopy. When this occurs will depend on environment and planting density but peak yield for example has been stated as occurring between 3-5 years after planting (Defra, 2007).

### 4.4 Survival and overwintering

Establishing a complete crop is more likely to impact yield for much longer since reduced spreading of the rhizome is considered an important characteristic to restrict invasiveness (Clifton-Brown et al., 2019) and therefore the commercial crop may not be able to fill any significant gaps if plant survival is less than 100% (Shepherd et al., 2020). As a consequence, the percentage of surviving plants is of greatest concern to researchers and growers because gaps can be expensive to remedy. Gaps can be detrimental to surrounding plant growth by allowing weeds to establish which may require higher management inputs.

Gaps may result from poor establishment and/or poor overwinter survival. Over winter survival is a factor of much concern among *Miscanthus* breeders, because wild *Miscanthus* used in breeding are often tropicalized species, and as such young, immature plants are at risk of over winter death, particularly if senescence and winter dry down has not been successfully completed (Tejera et al., 2019). Over wintering survival of several grass species grown in variable pot sizes and growth media, was assessed by Meyer and Cunliffe (2004) in Minnesota. One of these species was *Miscanthus sinensis* (Variegatus). The study concluded that container size had a significant effect on plant growth characteristics but not overwintering ability, and that *Miscanthus sinensis* had one of the lowest winter survival rates, leading them to conclude that genetic variability is a stronger factor in winter survival than plant size or growth medium. Our results add nuance to this conclusion in that there was a significant interaction between plug size and planting date on survival. When comparing the success of the two smaller modules with the two larger modules under the later planting condition, an observation of great commercial importance is the successful establishment of the larger modules. This demonstrated a significant impact of plug type on survival and the potential to extend the planting window, without negatively affecting the plant survival.

### 4.5 Length of time spent under glasshouse conditions

The length of time spent in plugs could potentially be a contributing factor to establishment differences seen but the time grown under glasshouse conditions also represents an additional cost. It was hypothesised that additional growth under the relatively superior growth conditions in a glasshouse compared to field conditions in year one would generate a superior crop. In fact the converse was true but probably for similar reasons to those discussed for the plug type trial and in general the sowing and planting trial was consistent with the plug type and planting trial. In the former trial a significant effect on establishment was noted as a result of planting date, but not the sowing date, suggesting age and morphology of the plantlets was less important than the environmental variables at planting. The second planting time was late June and produced significantly lower establishment rates than the first planting time in April. Harvestable yield was also affected by planting date and year of harvest, but not by sowing date. Also consistently across the two trials significant differences were lessened by the time of harvest after the second growth year, suggesting no long term residual effect is likely once the plantation has achieved economically harvestable yield. This makes it especially important to focus more on establishing full swards during the first year, than to focus on early yield.

Overall, the effects of module treatments under glasshouse conditions were significant but were less significant on field performance of *Miscanthus* than were the effects of the date of planting, which produced larger differences on the establishment and growth. This is consistent with a study based on establishment of Pine seedlings that described a hierarchy model illustrating the importance of factors affecting survival of transplanted pine seedlings (South, 2000). The most important factor in the model was the environment which included a range of variables such as soil type, meteorological variables, weed competition and herbivory. The second factor in the model the handling of the plants prior to planting which included cold storage length and temperature, depth of planting, and post planting care (for example applications of mulch films). The third and fourth factors were seedling morphology and seedling physiology, which were affected by nursery growth conditions. Our results are consistent with the hierarchy model of factors impacting the survival of transplanted pine seedlings (South, 2000). However, our study demonstrates the significant impact of glasshouse treatments on establishment under more challenging environments which may be important when growing *Miscanthus* in more marginal land or if there is a need for a longer planting window.

## 5 Conclusions/summary

The module size that provided the best balance of increased rooting depth and soil volume, with good survival and growth under most field conditions, and produced more plugs per unit area in the glasshouse, is the 45cm^3^ module (plug type 144). The largest module (70cm^3^, plug type 104) provided comparable survival and growth rates in field conditions, despite a significantly larger above and below ground biomass, but with an approximately 28% reduction in the number of plugs produced per unit area.

Large plug volumes allowed plants to mature more rapidly, reducing glasshouse time and also provided a longer planting window. Sowing date did not have a significant effect on establishment or any growth measurements including yield. This is an important outcome from our study since contrary to expectations additional time growing under more optimal controlled growth conditions had little impact. Sowing later improved plug production efficiency (**Table 1**), through reducing input requirements such as heat and light, therefore will not only reduce the carbon footprint of plug production but also reduce the cost of production.

The field conditions are most significant, and we suggest that optimal planting conditions involve a period of rain beforehand, and warm temperatures after planting. Mulch film covering provides plugs with greatly improved chances of survival after planting, but under high temperatures and direct sunlight can increase the risk of desiccation in weaker plugs. However, it is important that *Miscanthus* plants be able to establish under a variety of conditions. The results presented strongly suggest that late planting times should be avoided where possible, as the growth period in field was not sufficient to develop and mature to a high yielding and developmentally mature sward, regardless of initial biomass. Most importantly, later planting is more likely to coincide with higher temperatures and low soil water availability. The May planting appeared, in this study, to be the optimal planting time, with the best environmental conditions.

## Data availability statement

The raw data supporting the conclusions of this article will be made available by the authors, without undue reservation.

## Author contributions

CA, RW, PR, JC-B: Conceptualisation. MM: resources. CA, RW: Investigation. PR: Formal analysis. RW, CA, PR: writing original draft. JC-B, PR: funding acquisition. All authors contributed to the article and approved the submitted version.
